# Sorbitol induces apoptosis of human colorectal cancer cells via p38 MAPK signal transduction

**DOI:** 10.3892/ol.2014.1994

**Published:** 2014-03-24

**Authors:** XUE LU, CHUN LI, YONG-KUN WANG, KUN JIANG, XIAO-DONG GAI

**Affiliations:** 1 Department of Pathology, School of Basic Medical Sciences, Beihua University, Jilin, P.R. China; 2 Department of Pathology, Junan Hospital, Changchun, Jilin, P.R. China; 3 Department of Surgery, China-Japan Union Hospital of Jilin University, Changchun, Jilin, P.R. China

**Keywords:** sorbitol, colorectal cancer, apoptosis, mitochondrial death cascade, p38 mitogen-activated protein kinases

## Abstract

Sorbitol has been reported to have anticancer effects in several tumor models, however its effects on colorectal cancer remain elusive. In the present study, the effects of sorbitol on growth inhibition and apoptosis in the colorectal cancer HCT116 cell line were evaluated and its mechanism of action was examined. An MTT assay was utilized to determine the effect of sorbitol on HCT116 cell proliferation at different time points and variable doses. Western blot analysis was used to examine the effect of sorbitol on apoptosis-related protein expression and the p38 MAPK signaling pathway. The results revealed that sorbitol may inhibit the growth of HCT116 cells in a time- and dose-dependent manner. Following treatment with sorbitol for 3 h, western blotting demonstrated cleavage of the caspase-3 zymogen protein and a cleavage product of poly (ADP-ribose) polymerase (PARP), a known substrate of caspase-3, was also evident. During sorbitol-induced apoptosis, the mitochondrial pathway was activated by a dose-dependent increase in Bax expression and cytochrome *c* release, while the expression of anti-apoptotic protein Bcl-2 was significantly decreased in a dose-dependent manner. The investigation for the downstream signal pathway revealed that sorbitol-induced apoptosis was mediated by an increase in phosphorylated p38 MAPK expression. Overall, the observations from the present study imply that sorbitol causes increased levels of Bax in response to p38 MAPK signaling, which results in the initiation of the mitochondrial death cascade. Therefore, sorbitol is a promising candidate as a potential chemotherapeutic agent for the treatment of colorectal cancer HCT116 cells.

## Introduction

Colorectal cancer (CRC) is one of the most common malignant tumor types of the digestive tract. The incidence of CRC has been rising over the past several decades due to improving living standards and dietary changes. Clinically, due to preoperative medical imaging examination, surgical technology and the use of powerful chemotherapeutics, the prognosis for CRC has markedly improved. Despite this, CRC remains responsible for >10% of all cancer-associated mortalities (1). The occurrence of CRC is a complex multi-stage process and includes the disruption of intestinal epithelial cell proliferation, differentiation, apoptosis and survival (2). Abnormal signal transduction networks are involved in all stages of tumor development. A number of studies have demonstrated that abnormal cell proliferation and dysregulated apoptosis were correlated with the occurrence of human colorectal carcinoma (3). A variety of factors may decrease tumor cell apoptosis, acting through different signal transduction pathways, and resulting in CRC occurrence. The p38 mitogen-activated protein kinases (MAPK) signaling pathway is an important component of the MAPK superfamily, which is activated by diverse extracellular stimuli and has a central role in cell apoptosis. Abnormalities in this pathway are associated with tumorigenesis and the development of other proliferative diseases (4–7). Examining the role of the p38 MAPK signaling pathway in CRC development, using chemical intervention, may provide a reliable theoretical basis for further elucidating CRC pathogenesis.

Sorbitol has been used as a treatment for cerebral edema and glaucoma, and edematous oliguria of normal cardiac function, due to its property of diuresis dehydration. Recently, a number of studies have demonstrated that sorbitol may activate the p38 MAPK signal transduction pathway and induce the apoptosis of tumor cells (8–10). Therefore, it is important to examine further whether sorbitol is able to induce apoptosis of human colorectal cancer cells and to elucidate the associated molecular mechanisms.

The aim of the present study was to document the possible inhibitory effect of sorbitol in colorectal cancer, using the human colorectal cancer HCT116 cell line as a model system. Then, it was investigated whether the apoptosis and p38 MAPK signaling pathway described above were involved in sorbitol-induced HCT116 cell death.

## Materials and methods

### Reagents and antibodies

Sorbitol and MTT were employed (Sigma, St. Louis, MO, USA) as well as antibodies against tubulin, poly (ADP-ribose) polymerase (PARP), cleaved PARP (Santa Cruz Biotechnology, Inc., Santa Cruz, CA, USA), capase-3, cleaved capase-3, Bax, Bcl-2, cytochrome *c* (Abcam, Cambridge, UK), p38 and p-p38 (BD Pharmingen, San Diego, CA, USA).

### Cells and culture conditions

The colorectal cancer HCT116 cell line (Shanghai Institute of Cell Biology, Chinese Academy of Sciences, Shanghai, China) was maintained in RPMI-1640 medium (Gibco, Carlsbad, CA, USA). The medium was supplemented with 10% fetal calf serum (Gibco). A total of 100 U/ml penicillin and 100 μg/ml streptomycin (Gibco), and all of the cells were maintained at 37°C in a humidified atmosphere containing 5% CO_2_ and 95% air.

### MTT assay

Cell proliferation assay was determined by the conversion of MTT into water-insoluble formazan by viable cells. Briefly, HCT116 cells were seeded in 96-well microtitre plates with 1×10^4^ cells/well and incubated for 24 h in the same conditions. Then, the cells were treated with 0.5, 1.0 and 1.5 M of sorbitol for 3 h or with 1.0 M of sorbitol for 1, 2 and 3 h. MTT was added to the cells, which were then cultivated for a further 4 h. Following the removal of the supernatant fluid, 100 μl/well dimethyl sulfoxide (Gibco) was added to the cells, which were agitated for 15 min. The absorbance was measured at 570 nm by the enzyme immunoassay instrument (Bio-Rad 2550; Bio-Rad, Hercules, CA, USA) The untreated HCT116 cells served as the controls. Each assay was repeated three times. Cell viability was calculated as a percentage of the viable cells in the sorbitol-treated group versus the untreated control by following the equation: Cell viability (%) = [optical density (OD) (sorbitol)-OD (blank)/OD (control)-OD (blank)] × 100.

### Western blotting analysis

Following treatment of the cells with different concentrations of sorbitol (0.5, 1.0 and 1.5 M) for 3 h, PARP, cleaved PARP, capase-3, cleaved capase-3, Bax, Bcl-2, cytochrome *c*, p38 and p-p38 expression was detected by western blotting. A total of 1×10^6^ cells were washed with ice-cold phosphate-buffered saline (PBS; Sigma) twice and lysed with cell lysis buffer at 4°C for 30 min. Cell debris was removed by centrifugation at 15,000 × g for 15 min at 4°C. Equal amounts of proteins were separated by 10% SDS-PAGE and transferred onto nitrocellulose membrane. The membranes were first stained to confirm the uniform transfer of all samples and then incubated in the blocking solution for 2 h at room temperature. The membranes were first incubated with the primary monoclonal antibodies at a dilution of 1:1,000 for 2 h, followed by extensive washing with PBS twice and Tris-buffered saline with Tween 20 (TBST; Sigma) twice. The membranes were then incubated with their corresponding peroxidase-conjugated secondary antibodies (dilution, 1:1,000) and washed with TBST. Tubulin was used as an internal control. The immunoreactive proteins were detected using an Amersham ECL western blotting detection system (GE Healthcare, Amersham, UK).

### Statistical analysis

Statistical analysis was conducted using SPSS 11.5 software (SPSS, Inc., Chicago, IL, USA). All results are presented as the mean ± SD. The Student’s t-test was used for statistical analysis. P<0.05 was considered to indicate a statistically significant result.

## Results

### Sorbitol inhibits HCT116 cell proliferation

**Figure 1:**
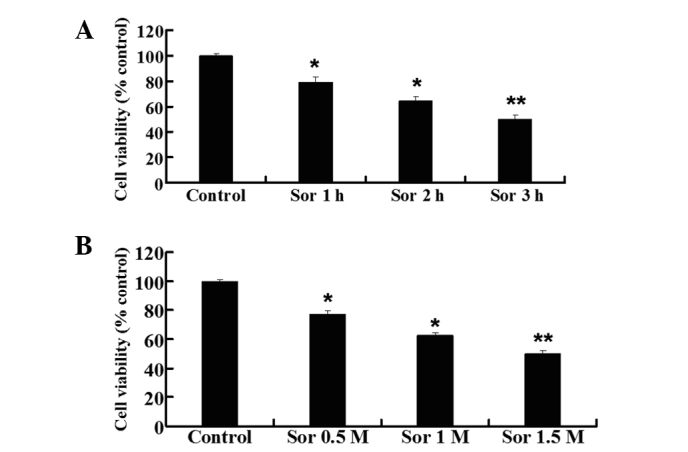
Cytotoxicity effects of sorbitol on HCT116 cells by the MTT assay. (A) HCT116 cells were treated with 1.0 M of sorbitol for 1, 2 and 3 h. (B) HCT116 cells were treated with sorbitol (0.5, 1.0 and 1.5 M) for 3 h. ^*^P<0.05 and ^**^P<0.01 as compared with the control group.x

To investigate the growth inhibitory effects of sorbitol on colorectal cancer HCT116 cells, the cells were treated with 1.0 M of sorbitol for 1, 2 and 3 h or with various concentrations of sorbitol (0.5, 1.0 and 1.5 M) for 3 h. As demonstrated in Fig. 1A, treatment of HCT116 cells with 1.0 M concentrations of sorbitol for 1, 2 and 3 h markedly inhibited cell growth in a time-dependent manner. The growth inhibitory effect of sorbitol on cells gradually increased with the duration of time (P<0.05). As revealed in Fig. 1B, sorbitol (0.5–1.5 M) also exhibited significant growth inhibitory effects on HCT116 cells in a dose-dependent manner. Sorbitol with concentrations of 0.5–1.5 M demonstrated markedly higher growth inhibitory effects than the control group (P<0.05).

### Sorbitol inhibits apoptosis-related protein expression

**Figure 2:**
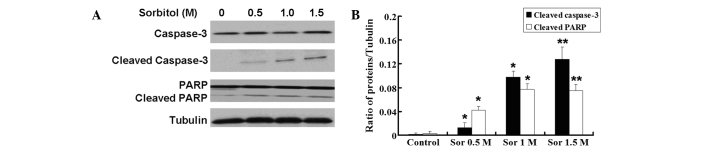
Expression of apoptosis-related proteins following sorbitol treatment. (A) Western blot analysis of caspase-3, cleaved capase-3, PARP and cleaved PARP. (B) Bar graphs represent the ratios of cleaved capase-3/tubulin and cleaved PARP/tubulin. ^*^P<0.05 and ^**^P<0.01 as compared with the control group. Sor, sorbitol; PARP, poly (ADP-ribose) polymerase.

The HCT116 cells were incubated with different doses of sorbitol (0, 0.5, 1.0 and 1.5 M) for 3 h, respectively. Following protein extraction, western blotting was used to examine the expression of apoptosis-related proteins, capase-3, cleaved capase-3, PARP and cleaved PARP. The results demonstrated that the expression of cleaved capase-3 and cleaved PARP were markedly increased in the sorbitol treatment group as compared with that in the control group (Fig. 2A), and this difference was statistically significant (P<0.05; Fig. 2B).

**Figure 3:**
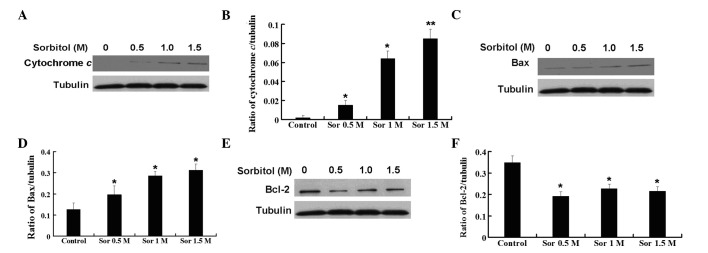
Sorbitol induces mitochondrial events associated with apoptosis in HCT116 cells. (A) Western blot analysis of cytochrome *c*. (B) Bar graph represents the ratio of cytochrome *c*/tubulin. (C) Western blot analysis of Bax. (D) Bar graph represents ratio of Bax/tubulin. (E) Western blot analysis of Bcl-2. (F) Bar graph represents ratio of Bcl-2 /tubulin. ^*^P<0.05 and ^**^P<0.01 as compared with the control group. Sor, sorbitol.

To estimate the contribution of the mitochondrial apoptosis-related pathway, the protein expression of cytochrome *c*, Bax and Bcl-2 were also detected by western blotting analysis (Fig. 3A, C, E). The results revealed that sorbitol significantly increased the expression of cytochrome *c* and Bax in HCT116 cells compared with that in the control group, while the expression levels of anti-apoptotic protein Bcl-2 were significantly decreased. These differences were statistically significant (P<0.05; Fig. 3B, D, F). The results suggest that sorbitol may induce apoptosis of HCT116 cells through the mitochondrial apoptotic signaling pathway.

### Sorbitol induces apoptosis via the p38 MAPK signaling pathway

**Figure 4:**
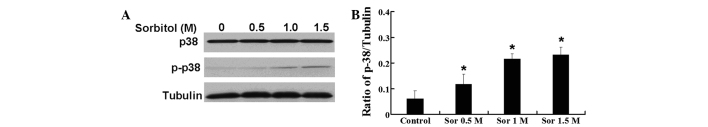
Sorbitol activates the p38 mitogen-activated protein kinase pathway in HCT116 cells. (A) Western blot analysis of p38 and p-p38. (B) Bar graph represents the ratio of p-p38/tubulin. ^*^P<0.05 and ^**^P<0.01 as compared with the control group. Sor, sorbitol.

Following treatment of HCT116 cells with different concentrations of sorbitol (0, 0.5, 1.0 and 1.5 M) for 3 h, the expression of p38 and p-p38 were analyzed by western blotting analysis. The results demonstrated that the expression of p-p38 was increased in the sorbitol-treated group (Fig. 4A). The difference was statistically significant compared with the control group (P<0.05; Fig. 4B). These results suggest that the activation of the p38 MAPK pathway may participate in the process of sorbitol-induced HCT116 cell apoptosis.

## Discussion

As an important raw material, sorbitol has been used extensively in the pharmaceutical, chemical, light and food industries. Sorbitol has also been used to treat cerebral edema and glaucoma, and edematous oliguria of normal cardiac function, due to its property of diuresis dehydration. A number of studies have demonstrated that sorbitol may activate the p38 MAPK signal transduction pathway and induce the apoptosis of tumor cells (8–10). In the present study, it was identified that sorbitol inhibited HCT116 cell growth and markedly induced apoptosis when the concentration of sorbitol was ≥0.5 M.

The total cell cycle time for numerous types of tumor is equal to or even longer than that of the corresponding normal cell (11–12). In healthy cells, there are certain restriction points, including the G1/S and G2/M stages. When DNA is damaged, the cell cycle is paused at a certain point, which facilitates cellular repair of such damage. When, however, DNA damage cannot be successfully repaired during the pause, the cells enter the apoptosis process by triggering the expression of apoptosis-induced genes. In tumor cells, there is no such repair function. Therefore, the balance between the proliferation and apoptosis of cells is disrupted and cell growth is unlimited by such repair mechanisms. According to these factors, chemotherapeutic agents aim to induce tumor cell apoptosis, block the cell cycle, directly kill tumor cells and induce the differentiation of tumor cells at certain sites and pathways of the cell cycle (13). However, in addition to high doses of chemotherapy and radiotherapy, one of the major antitumor effects of general antitumor drugs, radiation and hormone preparations is to induce the apoptosis of respective sensitive cells to achieve therapeutic purposes. Therefore, apoptosis has a critical role in cancer treatment (14).

In general, there are intrinsic and extrinsic apoptotic pathways, which differ in their activation of specific signaling pathways. The execution process of apoptosis is consistent and characterized by a distinctive series of biochemical events. One set of mediators implicated in apoptosis belongs to the aspartate-specific cysteine proteases or caspases (15). Their contributions are largely through the cleavage of a variety of substrates, including PARP, a known substrate of caspase-3. Thus far, >10 caspases have been identified. Caspase-3, as a critical apoptosis executive, is activated and the cleavage of its zymogen protein appears in the cytosol when apoptosis occurs (16). Bcl-2 family proteins are known to be involved in mitochondrial apoptotic pathway. The balance of pro-apoptotic (Bax, Bad and Bid) and anti-apoptotic members (Bcl-2, Bcl-xL and Mcl-1) regulates the responses of cells to apoptosis activators. The translocalization of apoptotic proteins from the cytosol to the surface of the mitochondria leads to the release of cytochrome *c* into the cytosol, where it is bound to apoptotic protease activating factor-1 and triggers the caspase cascade (17–19).

p38 MAPK, a main member of the MAPK family, is important in a variety of pathophysiological processes. Activated p38 MAPK may enter the nucleus, or be transferred to other cellular locations, and results in the activation of transcription factors when its serine/tyrosine residue is phosphorylated (20). It has been reported that p38 MAPK activity is associated with apoptotic induction in several cell types and in response to a multitude of cellular stresses (21). Activation of p38 MAPK is also required for numerous anticancer drugs to induce tumor cell apoptosis (22–24). At present, it is widely believed that p38 MAPK induces apoptosis involved with Bax translocation to the mitochondria and an increase in Bax/Bcl-2 ratio, cytochrome *c* release, and caspase-9 and caspase-3 activation (25–26).

In the present study, the data outline the antitumor mechanism of sorbitol treatment in colorectal cancer HCT116 cells. It was demonstrated that sorbitol induced apoptotic cell death, which was accompanied by the activation of caspase-3 and PARP (P<0.05). Further investigation revealed that sorbitol-evoked apoptosis occurred in colorectal cancer HCT116 cells via the mitochondrial death cascade, with an upregulation of Bax, downregulation of Bcl-2 and the release of cytochrome *c*. Furthermore, the results revealed that sorbitol was able to increase the level of phosphorylation of p38, which then activated the p38 MAPK pathway. In conclusion, the results demonstrate that p38 MAPK-mediated activation of pro-apoptotic protein Bax promotes sorbitol-induced apoptotic cell death via the mitochondrial death cascade. Therefore, sorbitol represents a promising candidate for the development of novel chemotherapeutic agents for the treatment of colorectal cancer.

## References

[1] Waldner M, Schimanski CC, Neurath MF (2006). Colon cancer and the immune system: the role of tumor invading T cells. World J Gastroenterol.

[2] Calvert PM, Frucht H (2002). The genetics of colorectal cancer. Ann Intern Med.

[3] Wang L, Xiao X, Li D (2012). Abnormal expression of GADD45B in human colorectal carcinoma. J Transl Med.

[4] Liu SI, Huang CC, Huang CJ (2007). Thimerosal-induced apoptosis in human SCM1 gastric cancer cells: activation of p38 MAP kinase and caspase-3 pathways without involvement of [Ca^2+^]i elevation. Toxicol Sci.

[5] Rabi T, Banerjee S (2008). Novel synthetic triterpenoid methyl 25-hydroxy-3-oxoolean-12-en-28-oate induces apoptosis through JNK and p38 MAPK pathways in human breast adenocarcinoma MCF-7 cells. Mol Carcinog.

[6] Park SJ, Kim IS (2005). The role of p38 MAPK activation in auranofin-induced apoptosis of human promyelocytic leukaemia HL-60 cells. Br J Pharmacol.

[7] Zhan Y, Gong K, Chen C (2012). P38 MAP kinase functions as a switch in MS-275-induced reactive oxygen species-dependent autophagy and apoptosis in human colon cancer cells. Free Radic Biol Med.

[8] Garmyn M, Mammone T, Pupe A (2001). Human keratinocytes respond to osmotic stress by p38 map kinase regulated induction of HSP70 and HSP27. J Invest Dermatol.

[9] Stadheim TA, Kucera GL (2002). c-Jun N-terminal kinase/stress-activated protein kinase (JNK/SAPK) is required for mitoxantrone- and anisomycin-induced apoptosis in HL-60 cells. Leuk Res.

[10] Stadheim TA, Saluta GR, Kucera GL (2000). Role of c-Jun N-terminal kinase/p38 stress signaling in 1-beta-D-arabinofuranosylcytosine-induced apoptosis. Biochem Pharmacol.

[11] Ribatti D, Vacca A (2008). The role of microenvironment in tumor angiogenesis. Genes Nutr.

[12] Zhao P, Zhong W, Ying X (2008). Manganese chloride-induced G0/G1 and S phase arrest in A549 cells. Toxicology.

[13] Johansson M, Persson JL (2008). Cancer therapy: targeting cell cycle regulators. Anticancer Agents Med Chem.

[14] Lee SK, Kim HN, Kang YR (2008). Obovatol inhibits colorectal cancer growth by inhibiting tumor cell proliferation and inducing apoptosis. Bioorg Med Chem.

[15] Salvesen GS, Riedl SJ (2008). Caspase mechanisms. Adv Exp Med Biol.

[16] Liu XD, Fan RF, Zhang Y (2010). Down-regulation of telomerase activity and activation of caspase-3 are responsible for tanshinone I-induced apoptosis in monocyte leukemia cells in vitro. Int J Mol Sci.

[17] Sun KW, Ma YY, Guan TP (2012). Oridonin induces apoptosis in gastric cancer through Apaf-1, cytochrome c and caspase-3 signaling pathway. World J Gastroenterol.

[18] Reed JC, Miyashita T, Takayama S (1996). BCL-2 family proteins: regulators of cell death involved in the pathogenesis of cancer and resistance to therapy. J Cell Biochem.

[19] Chao DT, Korsmeyer SJ (1998). BCL-2 family: regulators of cell death. Annu Rev Immunol.

[20] Lee JC, Kumar S, Griswold DE (2000). Inhibition of p38 MAP kinase as a therapeutic strategy. Immunopharmacology.

[21] Ichijo H, Nishida E, Irie K (1997). Induction of apoptosis by ASK1, a mammalian MAPKKK that activates SAPK/JNK and p38MAPK signaling pathways. Science.

[22] Hui L, Bakiri L, Stepniak E, Wagner EF (2007). p38alpha: a suppressor of cell proliferation and tumorigenesis. Cell Cycle.

[23] Chiacchiera F, Simone C (2008). Signal-dependent regulation of gene expression as a target for cancer treatment: inhibiting p38alpha in colorectal tumors. Cancer Lett.

[24] Bradham C, McClay DR (2006). p38 MAPK in development and cancer. Cell Cycle.

[25] Van Laethem A, Van Kelst S, Lippens S (2004). Activation of p38 MAPK is required for Bax translocation to mitochondria, cytochrome c release and apoptosis induced by UVB irradiation in human keratinocytes. FASEB J.

[26] Mandal C, Dutta A, Mallick A (2008). Withaferin A induces apoptosis by activating p38 mitogen-activated kinase signaling cascade in leukemic cells of lymphoid and myeloid origin through mitochondrial death cascade. Apoptosis.

